# Indents

**Published:** 1915-11

**Authors:** 


### INDENTS

☞ **Brief Articles of Technical or Scientific Value will receive notice and comment. Contributions invited.**

*Burns from Acids.* If you get carbolic acid on any surface where it is not wanted, apply absolute alcohol at once. If hydrofluoric acid, apply a strong solution of bicarbonate of soda. Never use these acids without having readily at hand some agent to stop their action immediately in case of accident.—D. C., in *Review.*

*Sterilization of Root-Canal Instruments.* The pulp extractors I employ are fitted into slender aluminum handles. Before using they are boiled and placed in test tubes containing a 50 per cent. alcoholic solution of lysol. All bristles, reamers, and drills are also boiled, and kept in suitable containers in a like solution. Broaches and canal pluggers, however, are boiled, dried, and placed in the cabinet, for these may be sterilized without injury by passing them through a Bunsen flame immediately preceding their insertion in a canal. Such a treatment would be ideal in the case of all canal instruments, for everyone recognizes that the drawers of a dental cabinet are far from aseptic. All highly-tempered instruments, of course, such as bristles, pulp extractors, reamers, and drills, would immediately be ruined by coming in contact with a flame.—A. P. Lee, in *Dental Cosmos.*

*Do Away with Pumice.* All operations where you use pumice can be performed better and quicker, as well as cleaner, with the use of air-floated silicate. Use in the same manner as you use pumice but in smaller quantity.

*To Restrict the Flow of Solder.* In soldering gold, when it is desired to restrict the flow to a certain area with a sharp lead pencil draw a line around the desired area. The solder will not flow past the line.

*A Good Coating for Plaster Casts.* To four ounces of sulfuric ether add two ounces of collodion and two ounces of silver gloss. The latter may be obtained from dealers in painters' supplies, and is put up in one-ounce packages. Let the mixture stand for about forty-eight hours and shake well before using. Apply with a camel's-hair brush and keep in well corked bottle.—Dr. J. C. Hopkins, in *Pacific Gazette.*

*To Compensate for Shrinkage in a Large Gold Inlay.* Where a cast gold inlay is to be made for a cavity involving the mesial, occlusal and distal surfaces of a bicuspid or molar if there is any shrinkage the inlay will invariably show a defective line at the gingival margins. To overcome this, the gingival margins of the cavity should be quite freely beveled so that the inlay will cover them with a lap joint instead of a butt joint. Even if there is a slight shrinkage the thin lap of gold can be burnished down to the cavity margin so that when cemented the inlay will perfectly seal the cavity.—I. D., in *Review.*

*Responsibility of the Dentist in Maintaining Oral Health for His Patients.* Very few people keep their mouths as clean as they should or as clean as they think they do. The result is that they harbor vast numbers of germs which may find their way into the crypts of the tonsils, the gastrointestinal tract, the accessory sinuses, and finally breaking down the soft tissues of the mouth, establish there chronic foci of infection. The danger lies in the constancy of the bacterial supply and the strain which it imposes upon the defensive forces of the body. A clean and healthy mouth should be the end of every dental operation. No patient should ever be dismissed until his mouth is clean and he has been most carefully instructed in how to care for it himself. This is said, realizing that it is not always possible to do this, through inability to control all patients, and realizing also that it is the most neglected feature of general dental practice. The "dental hygienist," who thoroughly cleanses the mouths of his patrons, is rendering a far more valuable service than is the dentist, who, neglecting this, makes the most perfect restorations of gold and porcelain. This is the first lesson in the dentistry of the twentieth century.—W. D. Tracy, in *Journal A. S.*

*A Danger in Analgesia.* An unfortunate feature of analgesia is that it caters to the dependence and lack of stamina of the patient. I do not by any means argue for the infliction of pain to develop courage on the part of the individual. But I do assert that in most of the routine work of dentistry the pain need not be so great that it works an injury to the patient or calls for a systemic numbing of the nerves, and that the lure of this practice held out to people will in many instances tend to minimize their self-control, and unfit them for meeting some of the other emergencies of life when they are thrust upon them. If we could insure every individual that he would never be called upon to endure hardship of any sort there might be some justification for humoring his every whim, but this is against all human experience, and the pampering of people beyond a reasonable limit is demoralizing in every way. I am aware that I shall be misunderstood, and mayhap misquoted in venturing this statement, and so I must hasten to make it clear that I am an advocate of the utmost consideration and kindliness in the treatment of people when they are obliged to encounter disagreeable experiences such as the performance of dental operations. Indeed, my chief contention is that there has not been sufficient consideration and kindness in the past, a fact which in part is accountable for much of the dread of the dental chair. Had dentists always been considerate, careful, and skillful, there never would have been any demand for such a practice as analgesia for filling teeth, and my advice to the dentist of today is to go back to the old-fashioned tenet represented by painstaking care, kindness, and delicacy of manipulation. If he will couple these with a constant study of human nature, he will meet all the contingencies of everyday practice without resort to so radical a procedure as analgesia.—C. N. Johnson, in *Cosmos*.

*Main Points in Treatment of Pyorrhea.* * * * There are three certain, undisputed factors in the treatment of pyorrhea that never can be overlooked in its successful treatment.

1st. The most important symptom to overcome is trauma. All loose teeth that have occlusion are found to possess this symptom. The trauma must be reduced by grinding, and the tooth ligated in such a manner as to insure stable fixation. Until this has been done there will be no progress.

2nd. Hygiene must be taught and established as a habit. The mouth must be made clean.

3rd. All concretions must be removed and all necrotic root surfaces made surgically clean. This must be accomplished by ''planing,'' or ''curetting,'' according to the instruments employed.

When these three things are correctly accomplished, the case will be so far advanced in convalescence that the thought of injecting emetin will not enter the operator's mind.

There is no easy method of curing pyorrhea, but there are some thousands of cases cured each year by scrupulously conscientious operators, who know their subject and who have had sufficient zeal and ability to master the technique necessary to secure results. Cases cured in this manner *stay cured,* as any competent periodontist will attest.—P. R. Stillman, *Items of Interest.*

*More Time Needed in Using Mouth Washes.* The use of a toothwash does not approach the conditions of a laboratory test, though there can be little doubt that a good deal of germicidal work in the mouth is done by the vigorous application of the toothbrush, and it may be pointed out that the tongue may well be included in the process. To be effective, however, the action of all antiseptics takes time, according to the vitality of the organisms they encounter, and usually the tooth-brushing process does not occupy many seconds. This question of time-exposure is important, but it is very generally overlooked, and consequently the antiseptic treatment of the teeth falls short of that effectiveness which is realized in laboratory experiments. The toothwashing process should be more prolonged, and the antiseptic wash allowed to remain in contact with the teeth and gums for some minutes instead of seconds before finally washing the mouth clean of the antiseptic with plain water.—Editorial, *The Lancet.*

*New York's Need.* Part of the editorial function, besides "adorning a tale," is to point a moral. Has not the time come for a great dental dispensary and institution of dental research to be established within the city of New York? That the greatest city in the Western Hemisphere should stand today with no considerable foundation for the advancement of dental science is an anomaly which merits heavy and increasing reproach. Columbia University and the Presbyterian Hospital are about to create a new medical school and hospital involving the investment of about fifteen million dollars—the result probably to be the greatest medical center in the world; but as yet the man of great means and of great foresight has not discovered this opportunity to endow a dental institution under the same auspices, which immediately would rank pre-eminent in the dental world. We do not doubt this need will be seen and met in the not distant future.

With the Evan's Institute, the Forsyth Infirmary and now the Rochester Dispensary actually and potentially in being, we may well infer, from the inspiration of these great works, and from the vast fields of untouched possibilities in public dental service, that developments beyond calculation in importance, humanly speaking, soon will come to pass.—Ed., in *J. of A. S.*

*Cutting Sensitive Dentine.* "In the August issue Dr. L. H. Gilbert describes a method of cutting sensitive cavities with less pain, saying: 'The major part of the pain is produced by the frictional heating up of the bur'; and his method is to play a stream of cold water upon the bur while operating, the saliva ejector drawing off the surplus. Yes! Good! But there is a little more scientific way. My preceptor, Dr. R. H. Hofheinz, lectured to me twelve years ago in college regarding bur heat, and declared that sharp burs produced less heat than dull ones. Also that by occasionally dipping the bur in carbolic acid, and then using it in the cavity, we have the bur lubricated to the extent of preventing much friction, surely less than when using water, carbolic being an oleum. Moreover, the protoplasmic contents of the tubuli which conduct the pain being coagulated by the carbolic, we reduce the sensitiveness to the minimum, have our field of operation constantly sterile, the infected area well disclosed by the carbolic, and all the time be freer in our work than if using a spray, causing water to splash all around.''—F. J. Shaddock, in *Items of Interest.*

*Obtunding Dentine.* ''After reading Dr. Gilbert's method of using a stream of cold water upon the bur, I think that would be a very safe place to use it, for I am confident if it was put into the cavity of some of my patients the landlord would have to repair the roof; besides I see no necessity of keeping the bur cool. A sharp bur at high revolution and the impact no harder than is necessary to cut and clear will prevent the bur from heating. To the best of my knowledge I am the first man in this country to run a dental engine by electricity successfully, and have used as high as 7,500 revolutions a minute, and, as the saying goes, 'The higher the fewer.' For excavating sensitive dentine I found this to be a very practical degree of speed, but pretty hard upon the hand-piece and difficult to obtain in the motors manufactured today. I had mine wound especially for 5,000 revolutions, which is considerably higher than those generally in use. I find there are a few men who appreciate the value of high speed and trust these words will quicken a few others to experiment with it for the benefit of the suffering public.—F. Bliven, in *Items of Interest.*

*Obtunding Dentine.* ''In view of the fact that some interest will undoubtedly be aroused in obtunding pastes by your publishing of Dr. Buckley's paper in the December 'Items of Interest,' I would like to call attention to a formula that I worked out four years ago and have been using in my practice with great success ever since. I communicated the formula to the San Francisco District Dental Society in open meeting on May 12, 1913—a year and a half before Dr. Buckley's paper appeared. It was published in the *Pacific Dental Gazette* of September, 1914. This, you will observe, was still thirty days before Dr. Buckley's address before the New York Society. In the meanwhile, the formula had been offerd to Dr. Kirk, of the *Cosmos*, for publication on July 23, 1914. The formula is as follows:

| | |
|---|---|
| Urea Hydrochloride and quinine | gr.20 |
| Paraformaldehyde | gr.15 |
| (The latter should be 20 grains instead of 15 grains if strong action is desired.) | |
| Thymol. | gr.20 |
| Zinc Oxide | gr.60 |

"A little of the 'putty' is sealed in the cavity for from one to three days, after which the tooth is quite insensitive. It will be seen that this is very similar to the mixture used by Dr. Buckley. It does not conflict with the Harrison Narcotic Law and has the great advantage of cheapness. Time and experience will have to decide whether the substitution of neothesin for quinine urea is of any advantage."—Joseph Novilsky, in *Items of Interest.*

*Acute Ulcerous Gingivitis.* This disease first described by Dr. Thomas L. Gilmer in the *Dental Review* of May, 1906, seems to be somewhat on the increase. At the time Dr. Gilmer wrote he made the statement that it was "seen but rarely," and in a relative sense this may still hold true, but it would seem in recent years to be more common than formerly. Up to the time of Dr. Gilmer's article the present writer recalls having seen only two, or possibly three, cases—the third having been so mild as not to be wholly typical—but within the past year at least four cases have come under his notice.

To quote briefly from Dr. Gilmer's article: "The onset of the disease is sudden, the earliest symptoms indicated by a slight malaise which is quickly followed by rapid ulceration, at first confined to the gingivae, usually about two or three of the anterior teeth on both jaws simultaneously and in corresponding localities; later it is extended to the gums about a number of the teeth, or groups of teeth, but rarely if ever does it include the entire gum margin."

In some cases the ulceration is quite severe, and the gums are excessively sensitive. For treatment Dr. Gilmer recommends antiseptic mouth washes such as warm boric acid solution, a cleansing of the surfaces with three per cent. pyrozone followed by the application of compound tincture benzoin. As a constitutional treatment he suggests four tablets of mercurous chlorid, 4-20 grain each, morning, noon and night, followed by a saline cathartic on rising.

The writer has found as a local application, following the thorough cleansing of the parts with an antiseptic spray driven by compressed air, the crystals of argyrol to be very effective. To place a small crystal less than the size of the head of an ordinary pin in each interproximal space is to see the crystals melt down and flow into every interstice about the teeth. This seems to aid materially in clearing up the affection, and hastening recovery.

It is hoped that the profession will be on the alert for this disease, and that ultimately we shall be better informed as to its etiology and thus better equipped to cope with it.—C. N. Johnson, in *Review*.

